# Nutrition in the Management of ADHD: A Review of Recent Research

**DOI:** 10.1007/s13668-023-00487-8

**Published:** 2023-07-28

**Authors:** Klaus W. Lange, Katharina M. Lange, Yukiko Nakamura, Andreas Reissmann

**Affiliations:** 1 Faculty of Human Sciences, University of Regensburg, 93040 Regensburg, Bavaria, Germany; 2 Department of Psychology, University of Bath, Bath, BA2 7AY Somerset UK

**Keywords:** ADHD, Management, Nutrition, Micronutrients, Minerals, Magnesium, Iron, Zinc, Vitamins, Vitamin D, Probiotics, Dietary patterns, Mediterranean diet, DASH diet, Few-foods diet, Oligoantigenic diet, Elimination diet, Lifestyle

## Abstract

**Purpose of Review:**

Various nutrients and diet quality have been suggested to be involved in the pathophysiology of ADHD. The purpose of this review was to examine data from recent cohort studies and dietary interventions to determine whether nutrition may play a role in the management of ADHD.

**Recent Findings:**

Preliminary evidence suggests that minerals might have beneficial effects on ADHD symptomatology. Probiotics might offer novel strategies to prevent or treat ADHD. Inverse associations between adherence to “healthy” diets and ADHD symptoms have been observed. Children with ADHD responding to the few-foods diet (or oligoantigenic diet) with an elimination of individually identified food items show substantially improved behavior and cognitive functioning.

**Summary:**

Evidence from recent research does not allow any recommendations regarding the use of micronutrients or probiotics in the management of ADHD. The few-foods diet may become an additional therapeutic option for children with ADHD.

## Introduction

Attention-deficit hyperactivity disorder (ADHD) is one of the most common psychiatric diagnoses in children and adolescents and is also increasingly diagnosed in adults [1]. ADHD is complex, heterogeneous, and multifactorial and is associated with widely diverse profiles of behavior, cognition, emotion, and motivation. The main symptoms of ADHD include age-inappropriate levels of inattention, impulsivity, and hyperactivity [1]. Comorbidity of ADHD with other mental conditions is frequently observed [2]. Genetic susceptibility interacting with social and environmental factors is assumed to contribute to the etiology of ADHD. Structural or functional brain changes cannot be found consistently in people with ADHD, and biological diagnostic markers underpinning the validity of the ADHD concept are lacking [3].

While short-term improvements of inattention, hyperactivity, and impulsivity can be achieved in children and adolescents with a diagnosis of ADHD by medication with psychostimulants, behavior therapy, and parent management training, the degree of efficacy of these therapies remains a matter of debate [4]. Numerous trials have shown that pharmacotherapy administered for several weeks can produce statistically significant improvements of the main ADHD symptoms, as assessed using clinical rating scales, compared to placebo. However, the minimum difference in rating scale scores indicating an ecologically relevant effect is unknown. Thus, statistically significant though small effects on symptom scores may not equate to clinically relevant improvements in difficulties in daily life [4].

The administration of drugs such as methylphenidate almost certainly yields some short-term symptom reduction in children and adolescents with ADHD. However, the extent of therapeutic efficacy is unclear due to the low quality of outcome measures and possible bias of trials. While randomized controlled trials supporting long-term effectiveness of psychostimulants for more than a few months are lacking [4, 5], the findings of observational studies have suggested potential long-term benefits of medication on serious problems co-occurring with ADHD, such as substance abuse, transport accidents, criminal convictions, and suicidal behavior [4]. Little is known about the safety of extended administration of psychostimulants. The findings of long-term observations suggest that children with a diagnosis of ADHD who were treated with psychostimulants into adulthood may present with a decrease in height as adults without any ongoing improvement in symptomatology [6].

Various lifestyle factors, including diet and nutrition, have been proposed to be involved in the pathophysiology and management of ADHD [7–9]. Emerging evidence points to a role of nutrition in brain development and functioning. Furthermore, various nutrients and diet quality have been linked to behavioral, cognitive, and affective functions as well as to the prevalence of mental disorders [10–12]. Nutritional deficiencies have been suggested to play a role in the pathophysiology and management of various mental disorders, such as depression, schizophrenia, autism spectrum disorder, and ADHD [13•, 14]. Nutritional approaches to the management of ADHD have therefore attracted increasing scientific interest [8, 15]. In recent years, several articles and chapters have reviewed the available scientific literature on ADHD and food, diet, and nutrition [16–21]. The current review provides an update of the most recent developments in this field.

## Search Strategy and Selection Criteria

Electronic search of PubMed was conducted to find original research articles, cohort studies, interventional trials, systematic reviews, and meta-analyses published from 2018 to 2022. Boolean search was conducted using (“nutrition” OR “food” OR “diet” OR “supplementation” OR “vitamin” OR “mineral” OR “fatty acid”) AND (“attention-deficit hyperactivity disorder” OR “ADHD” OR “attention deficit”) AND (2018/01/01:2022/12/08[dp]). A total of 873 papers were retrieved. Following title and abstract screening, duplicate studies, animal studies, articles without a special focus on ADHD and nutrition, papers published in journals without peer review, editorials, letters, notes, and conference abstracts were removed. According to these criteria, 54 studies examining the association of ADHD with nutrition were included in the present review.

## Micronutrients

A sufficient supply of micronutrients, such as minerals and vitamins, is required for normal brain development. Micronutrient deficiencies may contribute to dysfunctioning of the prefrontal cortex and other brain regions believed to play a role in the pathophysiology of ADHD [3]. Associations between symptoms of ADHD and mineral and trace element status regarding magnesium, iron, zinc, copper, and selenium have been proposed. However, the findings of studies exploring such associations have been inconclusive [22].

Hair trace element and mineral levels were examined in 52 boys with ADHD and 52 typically developing boys [23]. Statistically significant reductions in hair copper, magnesium, and manganese were found in children with ADHD. In regression models, hair zinc and magnesium levels were inversely associated with the severity of ADHD [23]. Serum mineral and trace element levels were measured in 68 children with ADHD and 68 typically developing children [24]. In comparison with controls, serum levels of chrome, magnesium, and zinc in children with ADHD were reduced by 21%, 4%, and 7%, respectively, while serum copper/zinc values were 11% higher [24]. The role of these changes in the pathophysiology of ADHD remains to be investigated.

Further studies on magnesium, zinc, and iron in ADHD have been published recently.

## Magnesium

People with ADHD have been assumed to be deficient in magnesium due to a low intake or elevated requirement. For example, in a group of 44 boys aged 4 to 9 years with ADHD, hair magnesium content was decreased by 11% compared to a control group of 32 typically developing boys [25]. Evidence concerning the association between serum concentrations of magnesium and the diagnosis of ADHD is conflicting. A recent systematic review and meta-analysis evaluated the available observational studies on this issue [26]. On the basis of seven studies, random-effects meta-analysis showed that individuals with ADHD had lower serum magnesium concentrations than healthy controls. This finding supports the hypothesis of an association of ADHD with serum magnesium deficiency. However, the causal relationship between magnesium levels and ADHD remains to be established.

The findings of preclinical studies have suggested that the administration of L-threonic acid magnesium salt in rodents is associated with neurofunctional effects that may offer clinical benefits in the therapy of ADHD. In an open-label pilot study, 15 adults with ADHD of moderate severity received L-threonic acid magnesium salt for up to 12 weeks [27]. Almost half of the participants showed a significant improvement on the ADHD severity rating scales used. The small sample size as well as the lack of a placebo group limits the significance of these findings.

A randomized, double-blind, placebo-controlled clinical trial of 66 children with ADHD examined the effect of both magnesium (6 mg/kg/day) and vitamin D (50,000 IU/week) supplementation for 8 weeks [28]. The use of a strengths and difficulties questionnaire was used to evaluate behavioral functioning and mental health at baseline and at the end of the study. Following 8 weeks of intervention, significantly elevated serum levels of magnesium and 25-hydroxy-vitamin D3 were found in the children of the intervention group when compared with the control children. Furthermore, children receiving magnesium plus vitamin D showed significant reductions in conduct, emotional, and peer problems as well as total difficulties in comparison with the placebo group [28]. Further studies using larger sample sizes and ecologically valid outcome measures are needed to establish the clinical relevance of these findings.

## Zinc

Zinc deficiency has been suggested to increase the risk of ADHD, while sufficient zinc supply is thought to improve ADHD symptoms. Since various studies regarding the zinc status in ADHD yielded inconsistent results, a recent systematic review and meta-analysis attempted to estimate the association between ADHD and serum/plasma and hair zinc concentrations [29]. This meta-analysis including 22 studies (1280 individuals with ADHD and 1200 controls) found no statistically significant difference in hair and serum/plasma zinc levels between people with ADHD and controls. Sensitivity analysis in studies examining circulating levels of zinc showed that the exclusion of one study changed the overall effect, with circulating zinc levels being significantly lower in individuals with ADHD in comparison with healthy controls [29]. Further well-designed studies need to clarify the pathophysiological role of zinc in ADHD.

A systematic review and dose–response meta-analysis, including six double-blind randomized controlled trials with a total of 489 children aged 7 to 10 years with a diagnosis of ADHD (245 children in the zinc group and 244 in the control group), attempted to quantify the efficacy of zinc supplementation on clinical ADHD symptoms in children [30]. Daily doses of zinc supplementation ranged from 10 to 40 mg; intervention duration varied between 6 and 12 weeks. Four studies used zinc supplementation as an adjunct to methylphenidate, while zinc was administered alone in the other two studies. Compared to controls, a statistically significant effect of zinc supplementation was found on ADHD total scores but not on hyperactivity and inattention scores [30]. Dose–response analysis did not reveal any significant non-linear association between the dosage or duration of zinc supplementation and ADHD total scores. The greatest symptom reduction was found after longer durations of zinc administration. The certainty of the evidence was rated moderate to very low for all outcomes [30]. Furthermore, most trials included in the meta-analysis were conducted in Asian populations with moderate to high risk of zinc deficiency. Thus, the findings obtained cannot be generalized to other populations. Future well-designed, large-scale, well-designed randomized controlled trials are needed to establish beneficial effects of zinc supplementation in ADHD.

## Iron

Systemic iron status in individuals with ADHD has been explored in numerous studies. A systematic review of available literature has examined whether children with ADHD have reduced serum or brain iron concentrations in comparison with healthy children [31]. This review included 20 case–control studies measuring differences in iron levels (serum iron, serum ferritin, or brain iron) between individuals with ADHD and controls. A statistically significant difference between these groups was found in 10 of 18 studies assessing serum ferritin concentrations and in 2 of 10 studies analyzing serum iron levels. While the results of systemic iron levels were inconsistent, the three studies investigating iron concentration in the brain reported significantly reduced levels in the thalamus of children with ADHD [31]. The limited evidence provided suggests that brain iron rather than systemic iron concentrations may be a biomarker of the pathophysiology of ADHD in children. Large longitudinal magnetic resonance imaging studies are required to assess correlations of ADHD symptoms with iron deficiency in specific brain regions.

An association between iron deficiency in infancy (ages 12 and 18 months) and sluggish cognitive tempo or ADHD symptoms, as rated by the children’s mothers, in childhood and adolescence (ages 5, 10, and 16 years) was examined in 959 children in Chile [32]. After adjusting for various covariates, greater severity of infant iron deficiency in infancy was associated with more frequent symptoms of sluggish cognitive tempo and ADHD at all ages studied [32]. The long-term associations revealed between iron deficiency in infancy and ADHD behaviors suggest that neurodevelopmental alterations caused by postnatal iron deficiency might play a role in the pathophysiology of ADHD. Interventions supporting brain development following early nutritional deprivation may be beneficial.

A qualitative systematic review of nine randomized clinical trials examined the efficacy of iron-zinc supplementation in the treatment of children and adolescents with ADHD [33]. The results of this review indicated that low zinc and iron levels were associated with higher baseline levels of ADHD severity and poorer treatment outcomes. Dietary supplementation with zinc and iron showed improvements in ADHD symptom severity compared to placebo control. However, the effect sizes of outcomes were small and related to specific ADHD symptoms [33]. Iron-zinc supplementation may be helpful for subgroups of youths with ADHD.

## Vitamins

The findings of several observational studies have suggested a role of vitamin D in the pathophysiology of ADHD. The pooled data of a meta-analysis including eight observational studies (with 2655 children with a diagnosis of ADHD and 8669 healthy controls) showed statistically significant reduced 25(OH)D levels in children with ADHD compared to controls [34]. Large cohort studies are required to investigate whether vitamin D-deficient infants are more likely to develop ADHD in the future. A systematic review and meta-analysis of four randomized controlled trials with 256 children, examining the effects of vitamin D supplementation as adjunctive therapy to methylphenidate on ADHD symptoms, showed a small but statistically significant improvement in ADHD total scores, hyperactivity scores, inattention scores, and behavior scores [35]. However, these effects were limited by the low to very low quality of evidence provided by the available studies. Effects of combined supplementation of vitamin D and magnesium [28] are described above.

## Omega-3 Fatty Acids

Deficiencies in and supplementation of polyunsaturated fatty acids have been suggested to play a role in the etiology and therapy of mental disorders [10]. The association of prenatal omega-6/omega-3 fatty acid ratio in umbilical cord plasma with ADHD symptoms at 4 and 7 years of age has been evaluated in a population-based birth cohort [36]. Omega-6 (arachidonic acid) and omega-3 (eicosapentaenoic and docosahexaenoic acid) fatty acid concentrations were measured in cord plasma. The ADHD symptoms of 580 four-year-old children were assessed using teachers’ reports (DSM-IV ADHD criteria); ADHD symptoms were also assessed by parents (Conners’ rating scale-revised short form) when the children were 7 years old (*N* = 642). While a higher omega-6/omega-3 ratio in cord plasma was associated with a higher ADHD index at age 7 years, no association was found at 4 years. No associations were observed for ADHD symptom diagnostic criteria [36]. These results suggest that a maternal diet during pregnancy with a high ratio of omega-6 to omega-3 fatty acids may influence the risk of the development of (subclinical) ADHD symptoms in the offspring during childhood.

In recent years, various clinical trials have examined the efficacy of omega-3 supplements in children with ADHD. A randomized placebo-controlled trial, including 162 children and adolescents aged 6 to 15 years with moderate ADHD symptoms, evaluated the effects of supplements containing docosahexaenoic acid and eicosapentaenoic acid or of a placebo, which were administered for 3 months [37]. This trial did not show any beneficial effect of omega-3 supplementation, since the total score reduction in the ADHD-RS-IV was greater in the placebo group than in the omega-3 group [37]. The effects of dietary docosahexaenoic acid supplementation on behavior were assessed in a 6-month randomized, placebo-controlled clinical trial with a total of 50 drug-naïve children aged 7 to 14 years with ADHD [38]. This trial found no significant difference between the treatment groups in the ADHD rating scale IV after 4 and 6 months. A 6-month double-blind placebo-controlled randomized trial investigated the effects of dietary supplementation with docosahexaenoic acid on ADHD symptoms in 66 individuals aged 6 to 18 years with ADHD. Between-group differences in favor of the omega-3 trial arm were found for behavioral measures, as assessed using the abbreviated Conners’ rating scale [39]. These findings suggest some beneficial effects of omega-3 fatty acids on symptoms of ADHD. Following dietary omega-3 fatty acid supplementation for 8 weeks, impulsive behavior, as assessed using the Barratt impulsiveness scale adapted for children, was examined in a randomized clinical trial comprising children and adolescents aged 6 to 16 years with ADHD [40]. The supplementation group showed significantly lower impulsiveness scores after the intervention, while no changes were found in the control group.

The effects of omega-3/omega-6 fatty acid supplementation in 40 preschool children aged 3 to 6 years with elevated levels of ADHD symptoms were investigated in a 4-month randomized, double-blind, placebo-controlled trial [41]. Intention-to-treat analyses provided some evidence of positive effects of omega-3/omega-6 fatty acids on parent- and teacher-rated ADHD symptoms. The effects of an omega-3/6 dietary supplement versus placebo on inattentive symptoms in a total of 160 children aged 6–12 years with inattentive ADHD were examined in a randomized controlled trial with a 6-month double-blind evaluation period [42]. The response rates were similar in both groups after 6 months, and no clinical benefits of omega-3/6 supplementation were detected [42].

A systematic review of seven randomized controlled trials with a total of 926 participants examining the effects of omega-3 supplementation in children and adolescents with ADHD found no significant reduction in ADHD scores, as measured by Conners’ rating scales [43]. Thus, there was no evidence supporting efficacy of omega-3 supplementation in reducing the degree of ADHD symptoms. However, some supportive evidence was found in a systematic review and meta-analysis examining the effects of omega-3 fatty acids on clinical symptoms and cognition in children and adolescents with ADHD [44]. In seven randomized controlled trials (534 randomized youths with ADHD), omega-3 fatty acid supplementation significantly improved clinical symptom scores. In three randomized controlled trials (214 randomized youths with ADHD), omega-3 administration improved cognitive measures associated with attention [44]. A more recent systematic review and meta-analysis of 31 clinical trials including 1755 children and adolescents with ADHD found no effects of polyunsaturated fatty acid supplementation on ADHD core symptoms, behavioral difficulties, or quality of life [45•]. These findings do not suggest any benefits of polyunsaturated fatty acids in ADHD. However, the certainty of evidence was questionable.

In summary, the evidence of therapeutic efficacy of polyunsaturated fatty acids regarding ADHD core symptoms appears to be marginal or non-existent. Pre-treatment status of omega-3 fatty acids may influence the effects of supplementation, and therapeutically relevant effects may be confined to people with omega-3 deficiency. Potential adverse effects of long-term omega-3 fatty acid supplementation should be considered [46].

## Probiotics

Intestinal microbiota and probiotics may affect brain activity, behavior, and mental health [47, 48]. Therefore, gut microbiota and the gut-brain axis have also become a focus of interest with respect to the pathophysiology of ADHD [49]. The findings in animal studies suggest that the gut microbiota may be a potential target in the management of ADHD [50]. Microbiome shifts have been observed in people with ADHD compared to healthy individuals, with certain bacterial taxa being less and others more abundant [51]. However, because of the high variability in the cohorts examined, the very low number of individuals included, and methodological differences of microbiome analysis [52], relatively little is known of the role of different gut microbiota compositions in the predisposition for ADHD. Therefore, it is at present impossible to define microbiome biomarkers for ADHD or to describe the underlying microbiome-mediated pathophysiological mechanisms. Furthermore, the analysis of the taxonomic microbiota composition may be insufficient and should be complemented by including prevalent microbial molecular function or genetic differences at the sub-species level of bacteria [52].

Preliminary interventional evidence has suggested preventive and therapeutic efficacy of probiotics in ADHD [53]. More recently, a pilot double-blind randomized placebo-controlled trial examined the effect of the probiotic strain *Lactobacillus rhamnosus* GG ATCC53103 on ADHD symptoms and health-related quality of life in 32 drug-naïve children and adolescents aged 4–17 years with a diagnosis of ADHD [54]. Children and adolescents with ADHD who received supplementation with *Lactobacillus rhamnosus* reported significantly better health-related quality of life as well as physical, social, school, and emotional functioning after 3 months of treatment in comparison with placebo controls. This suggests that administration of *Lactobacillus rhamnosus* could be beneficial. However, the psychometric results of parent and teacher reports showed no clear benefits of probiotic supplementation [54]. Future trials should be conducted over a longer period and include more participants and also children who are not drug-naïve.

In conclusion, while probiotics may offer novel strategies in the prevention or treatment of ADHD, the role of intestinal microbiota in the pathogenesis of ADHD and of probiotics in the management of ADHD requires further robust evidence from high-quality, large-scale intervention trials targeting microbiota.

## Dietary Patterns

Certain dietary patterns, whole diets and other lifestyle-related factors, rather than single nutrients have been suggested to be helpful in the management of ADHD [13•]. Several cross-sectional and case–control studies have evaluated the role of dietary patterns in the management of ADHD [18].

The findings of several studies exploring potential associations between ADHD and dietary exposures have suggested adverse effects of increased intake of sucrose. A recent birth cohort study from Brazil has investigated the association between the consumption of sugar in children aged 6 to 11 years, as estimated using a food frequency questionnaire, and the presence of ADHD, as assessed by mothers with the development and well-being assessment [55]. No association was found between permanent high versus low sucrose consumption in 6–11-year-old children and the incidence of ADHD. Increased sugar consumption by children with ADHD may be a consequence rather than a determinant of the disorder.

A cohort study from Norway explored associations between the quality of the diets of mothers during pregnancy and of their children at age 3 years, as assessed using diet quality indices, and ADHD symptoms and ADHD diagnosis in the children, as assessed at age 8 years using a parent rating scale for disruptive behavior disorders and a patient registry, respectively [56]. On the basis of 77,768 mother–child pairs eligible for studying ADHD diagnoses and 37,787 pairs for ADHD symptoms, better overall maternal diet quality during pregnancy was found to be associated with a small reduction in the ADHD symptom score at 8 years and a lower risk for ADHD diagnosis. No associations were observed between child diet quality and ADHD symptoms or diagnosis [56]. These results do not allow causal inferences because of potential unmeasured confounding.

Nutrient intake and dietary patterns were assessed in a case–control study from Iran, comprising 200 children aged 5 to 13 years (100 children with a diagnosis of ADHD and 100 healthy peers) [57]. In comparison with the control group, children with ADHD were reported to consume significantly more simple sugars, ready-made meals, and tea and significantly less protein, vitamins B1 and B2, vitamin C, calcium, and zinc. Both body mass index and waist circumference of children with ADHD were significantly higher [57].

A study from Spain with 259 preschool children aged 3 to 6 years (57 with ADHD and 202 control children) examined dietary patterns of children with and without ADHD diagnosed according to DSM-5 [58]. Principal component analysis performed to analyze dietary patterns based on food consumption frequency revealed a healthy pattern and unhealthy patterns (western-like and sweet patterns). The ADHD group was found to have a significantly negative association with the healthy pattern and a significantly positive association with the western-like diet. Children with inattentive presentation showed a lower adherence (12.2%) to a healthy dietary pattern than those of the control group (39.9%). Adherence to the sweet dietary pattern was similar in both groups [58]. These findings suggest an association between ADHD and dietary habits, with inattentive children showing a particular risk of unhealthy eating.

Another study investigated the longitudinal association between dietary patterns in 4-year-old children and ADHD symptoms in the same children at age 6 years [59]. Dietary intake was estimated using a food frequency questionnaire with 33 food groups. Major dietary patterns were identified based on the consumption of sweets, vegetables, meats, and carbohydrates. Parent assessment of ADHD symptoms used the Korean version of an ADHD rating scale. A sweet dietary pattern was found to be associated with an increased risk of attention deficit, hyperactivity, and ADHD symptoms. A food item analysis of this dietary pattern showed that consumption scores for chocolate, chips, and fruit jams correlated positively with attention deficit, hyperactivity, and ADHD symptoms. In contrast, a vegetable dietary pattern was associated with a reduced risk of ADHD symptoms [59].

Data from a prospective nationwide cohort study conducted in Korea were used to investigate the relationship between changes in dietary intake and prevalence of ADHD in 1733 elementary school children with ADHD scores and information on dietary intake, as assessed both at baseline (phase 1) and 2 years later (phase 2) [60]. A notable finding within the group of children whose ADHD symptoms had improved in phase 2 was an increase in the intake of vegetable protein. A between-group comparison showed that significant changes in nutrient intake were found mainly in the group of children with a diagnosis of ADHD in phase 2, whose nutrient intake (e.g., total fat) tended to be higher than that in children with improved symptoms. Intake of total fat and animal protein showed a positive correlation with the prevalence of attention deficit, while vegetable iron, zinc, calcium, and vegetable protein showed negative correlations with ADHD symptoms [60]. These findings suggest that nutritional status should be considered to ameliorate ADHD symptoms in school-age children. Future studies investigating potential preventive effects of nutritional supplementation on ADHD symptoms should examine the optimal age and treatment period for such interventions.

A case–control study from Iran, conducted with a total of 360 children and adolescents (120 cases and 240 controls) aged 7 to 13 years, found that a higher dietary phytochemical score, indicating the percentage of daily energy intake from phytochemical-rich foods (fruits, vegetables, legumes, whole grains, nuts, soy products, seeds, and olive oil), was associated with a lower risk of ADHD, as diagnosed using the DSM-IV-TR [61]. This result is in accordance with previous findings demonstrating a reduced risk of ADHD in children with a dietary pattern rich in phytochemicals [62] and a high intake of fruits and vegetables [63, 64]. Clinical trials are required to establish a causal relationship between dietary phytochemicals and the risk of ADHD.

In a case–control study comprising a total of 400 preschool and school children aged 4 to 12 years (200 children with ADHD diagnosis and 200 healthy controls), a significant inverse association was observed between polyphenol intake, as assessed using a food-frequency questionnaire, and the presence of ADHD, as diagnosed according to the DSM-5 criteria [65].

The association between adherence to a Mediterranean diet containing vegetables, legumes, nuts, fruits, grains, and fish, as assessed using a food frequency questionnaire, and the odds of ADHD, as diagnosed using the DSM-IV, in primary school children aged 7 to 13 years was investigated in an age- and sex-matched case–control study from Iran [66]. After adjusting for several potential confounders, the children in the highest tertile of Mediterranean diet adherence had a lower odd of ADHD in comparison with those in the lowest. These results suggest a beneficial effect of adherence to a Mediterranean diet on the odds of ADHD in primary school children. The results of an 8-week cross-sectional dietary intervention study from Spain investigating the effect of a Mediterranean diet on the progression of impulsive behavior, as assessed using the self-administered Barratt impulsiveness scale, in 60 children and adolescents with ADHD aged 6–16 years were inconclusive [67].

Several components of the dietary approaches to stop hypertension diet, such as high amounts of fruits, vegetables, low-fat dairy products, and vitamin C and low amounts of simple sugars, have been suggested to improve ADHD symptoms. A randomized controlled trial investigated the effect of this diet administered for 12 weeks to 80 children with ADHD aged 6 to 12 years [68]. Following adjustment for confounders, various measures determined by the abbreviated 10-item Conners’ scale, 18-item Swanson, Nolan and Pelham scale and the strengths and difficulties questionnaire, parent-, teacher- and child-reported hyperactivity, emotional symptoms, teacher-reported conduct problems, peer relationship problems, and prosocial behaviors were significantly improved in the dietary approaches to stop hypertension group compared to the control group [68]. Further randomized controlled trials with longer follow-up periods and ecologically relevant outcomes are needed to confirm these findings.

The results of a systematic review and meta-analysis of six dietary pattern studies (*N* = 8816) suggest that a “healthy” dietary pattern, which is high in vegetables, fruits, seafood, polyunsaturated fatty acids, magnesium, zinc, and phytochemicals, is associated with a significantly reduced risk of ADHD, while the “western” dietary pattern, which includes large amounts of confectionery, red meat, processed meats, refined grains, fried potatoes, crisps, soft drinks as well as animal and hydrogenated fats, and the “junk food” pattern, consisting of biscuits, chocolate bars, buns, cakes, pizza, sweets, crisps, and fizzy drinks, are associated with an elevated risk [69].

The temporal direction of the association between dietary patterns and ADHD symptoms, i.e., whether diet predicts ADHD or vice versa, is unclear. In a prospective cohort from the Netherlands, including 3680 school-aged children, ADHD symptoms were examined at ages 6 and 10 years with a parent-report questionnaire (Child Behavior Checklist), and dietary intake was assessed at age 8 years using a validated food-frequency questionnaire [70]. Multivariable linear regression analysis showed that more ADHD symptoms at age 6 years were associated with a lower diet quality score at age 8 years, while diet quality at age 8 years was not associated with ADHD symptoms at age 10 years. Furthermore, cross-lagged models confirmed a unidirectional link from ADHD symptoms to diet quality but not vice versa [70]. These findings suggest that children presenting with more ADHD symptoms may be at an increased risk of an unhealthy diet but that overall diet quality does not affect the risk of ADHD. A low intake of various nutrients could result from decreased appetite as a consequence of ADHD medication.

Other lifestyle factors than nutrition may also play an important role in the management of ADHD. The question of whether individuals with and without ADHD differ from one another in regard to healthy lifestyle behaviors has been explored [71•]. The parents of children aged 7 to 11 years with very well-characterized ADHD (*N* = 184) and typically developing children (*N* = 104) completed a lifestyle questionnaire assessing water intake, consumption of sweetened beverages, use of multivitamins or supplements, reading, screen time, physical activity, and sleep. A lifestyle index score was formed from these domains. After adjustment for age, sex, intelligence quotient, use of ADHD medication, household income, and other comorbid mental conditions, children with ADHD were almost twice as likely to have fewer healthy behaviors [71•]. These findings suggest that future studies should investigate the effects of combined lifestyle interventions in individuals with ADHD [72].

## Few-Foods Diet

Recent evidence suggests that the investigation of the role of food hypersensitivities in ADHD is a promising avenue worthy of further exploration. In particular, a large randomized controlled study has sparked interest in the idea that food additives may trigger hyperactivity in children [73]. While several meta-analyses have shown statistically significant, but small adverse effects of artificial food colorants on ADHD symptoms, there is no convincing evidence of the efficacy of food color elimination in the treatment of ADHD [18]. Beneficial effects of excluding food colors may be limited to children selected for food sensitivities. Therefore, subgroups of people with ADHD who may benefit from the exclusion of food additives need to be identified.

Some children with ADHD have been hypothesized to show hypersensitivities or allergic reactions to a variety of food items [74], which has led to the development of the oligoantigenic or few-foods diet. The exclusion of many food items in a strict elimination diet has been shown to be of value in assessing whether ADHD symptoms are induced by individual foods [75]. The few-foods diet eliminates the majority of food items from the diet for a limited time period and subsequently adds single foods one at a time. The initial elimination of foods helps determine whether food triggers ADHD symptoms. Children responding to the few-foods diet show improved behavior or cognitive functioning after a number of weeks. Subsequently, food items are consecutively re-introduced in a controlled manner in order to ascertain which foods are causative of symptoms or adverse reactions. Finally, an individualized diet is composed eliminating the identified foods.

Several double-blind placebo-controlled studies examining the effects of the few-foods diet in children with ADHD have shown that foods can trigger ADHD, suggesting the existence of a food-related subtype of ADHD [76]. Moreover, a randomized controlled trial revealed significant effects of the few-foods diet in an unselected sample of children with ADHD [75].

In an uncontrolled, open-label dietary intervention study of a small sample of eight children aged 8 to 14 years with a diagnosis of ADHD according to ICD-10, food items which are commonly related to intolerances were eliminated for 4 weeks [77]. Five of eight children showed a significant symptom improvement of > 40% in the ADHD rating scale IV after the diet. In the following re-introduction phase lasting 8 to 16 weeks, nutrients with individual relevance to ADHD symptoms were identified. This study confirmed findings of previous research, with about 60% of patients showing a significant improvement after 4 weeks of consuming a few-foods diet. In addition, the study demonstrated a high inter-rater reliability between a non-blinded child and adolescent psychiatrist and three blinded raters who evaluated ADHD rating scale IV scores in a pseudonymized video rating [77]. These results call for further randomized controlled trials.

A further study attempted to reveal how foods that may impact ADHD symptoms can be identified [78•]. In this uncontrolled, open trial, 16 children with a diagnosis of ADHD were assessed before and after a restricted elimination diet. Participants kept a daily 24-h recall journal on nutrition and behavior and filled in the abbreviated Conners’ scale to identify foods which increased ADHD symptoms. After 4 weeks of elimination diet, the individual food sensitivities were identified in the re-introduction phase. A repetitive increase of ADHD symptoms by at least two points in the abbreviated Conners’ scale after food introduction was found to hint at food sensitivity. Twenty-seven different foods, including milk and dairy products, cocoa, peanut, grains, and corn, were found to increase ADHD symptoms. Most participants showed sensitivities to more than one food [78•]. The results suggest that the few-foods diet combined with subsequent food challenge is a valid method to identify individual food sensitivities in people with ADHD.

In order to examine the effect of a few-foods diet on physical complaints, such as headache, asthma, rhinitis as well as gastrointestinal and sleep problems, unpublished data from previously published studies have recently been analyzed [79]. Children with ADHD either followed a 5-week few-foods diet or received advice on healthy nutrition. A clinically relevant reduction for 10 of 21 complaints, as assessed using a physical complaint questionnaire, was found in the few-foods group compared to controls [79].

In the Netherlands, the approach using the few-foods diet in children with ADHD is applied in practice. A retrospective study, including data from all children who started the few-foods approach in three specialized healthcare facilities during three consecutive months, has assessed the effectiveness of the few-foods approach in ADHD and oppositional defiant disorder in real life [80•]. The findings of this study, including a total of 57 children, showed that the few-foods diet, administered in general practice by trained physicians for 5 weeks, may have clinically relevant effects on symptoms of ADHD and oppositional defiant disorder, both in children with and without medication for ADHD [80•]. The use of medication was significantly reduced in responders to the few-foods diet. Using this diet in practice may result in secondary prevention of ADHD.

A recent study investigated the long-term effects of a few-foods diet on ADHD symptoms [81••]. Twenty-eight children and adolescents aged 7 to 14 years with a diagnosis of ADHD according to the criteria of DSM-IV and ICD-10 received a few-foods diet for 4 weeks. Twenty-one participants were re-assessed after 3.5 years [81••]. Fourteen of these participants fulfilled the responder criterion. In comparison with the baseline before the dietary intervention, the mean ADHD rating scale IV score showed a significant improvement not only immediately after commencing the diet, but also at follow-up 3.5 years later [81••]. These results suggest that individually adjusted nutrition may offer a long-term improvement of ADHD symptoms.

The mechanisms underlying the reduction in ADHD symptoms following a few-foods diet are unknown. An open-label intervention study examined whether behavioral changes after a few-foods diet are associated with altered brain function during inhibitory control in 79 boys aged 8 to 10 years with ADHD [82]. According to parents’ ADHD rating before and after the few-foods diet, 50 (63%) of 79 participants were diet responders, showing a reduction of ADHD symptoms of at least 40%. Functional magnetic resonance imaging was performed during a stop-signal task before and after the few-foods diet. Region-of-interest analyses found that activation in brain regions implicated in the stop-signal task was not associated with changes in ADHD symptoms. However, whole-brain analyses demonstrated a correlation between the decrease in ADHD symptoms and an increase in precuneus activation [82]. These findings suggest that a neurocognitive mechanism may be involved in the effects of the few-foods diet in children with ADHD.

In summary, the available findings regarding the few-foods diet confirm the hypothesis that food intolerances are a possible cause of ADHD.

## Conclusion

Whether or not individuals with low concentrations of minerals, vitamins, or omega-3 fatty acids are more responsive to dietary administration is unclear. Subgroups of people with ADHD who are likely to benefit from micronutrient or omega-3 fatty acid supplementation need to be identified. Supplementation above a certain threshold of micronutrient status might be ineffective. Moreover, since various comorbid psychiatric conditions are very common in individuals with ADHD, the efficacy of nutritional supplements may vary depending on the comorbidities of trial participants. The question of whether the administration of certain nutrients is effective at any time during the life span or confined to critical time windows also remains to be investigated. Nutrition-based prevention of ADHD may need to focus on pregnancy and infancy. This should be examined in future investigations. Dosage of dietary supplements is a critical issue, since the high-dosage administration of seemingly healthy minerals, vitamins, and other nutrients may carry the risk of adverse effects. In addition, the ill-defined nature of ADHD and the lack of biomarkers underpinning the concept of ADHD may hinder the identification of the role of nutrients and diet. Given the complexity and heterogeneity of the multiple biological, environmental, and social factors underlying ADHD, a simple one-fits-all solution regarding micronutrients and diets in the management of ADHD is unlikely to exist.

While the findings of observational studies suggest a role of dietary patterns in the management of ADHD, the designs of these studies are unable to establish a causal relationship between ADHD and diet. Associations between a low prevalence of ADHD and adherence to healthy dietary patterns do not necessarily imply protective effects of the foods consumed in childhood. For example, the mothers of children consuming healthy diets may also have eaten healthy foods during pregnancy and have therefore provided their offspring with essential nutritional compounds during critical phases of brain development. The associations observed between ADHD risk and dietary habits may also be explained by reverse causation, with ADHD behaviors leading to a preference for certain foods. Furthermore, associations between diet and ADHD may be caused by other (possibly causal) factors, which were not assessed. For example, lifestyle factors such as physical activity may correlate with dietary habits and may be more important factors in ADHD symptomatology. Children with ADHD may benefit from generally improved lifestyle choices. Therefore, the interaction between nutrition and lifestyle should play a greater role in investigations of the management of ADHD.

Evidence from recent research does not suffice to recommend the administration of micronutrients, omega-3 fatty acids, or probiotics in the management of ADHD. However, emerging evidence suggests that subgroups of children and adolescents with ADHD may benefit from the elimination of certain foods. A major benefit of the few-foods diet is that it may be tailored to the individual. The high response rate to this diet, of up to 60% reported in several studies, indicates an important role of food intolerances in the pathophysiology of ADHD. This promising personalized nutrition-based approach to the management of ADHD deserves further systematic investigation and should be considered in all children with ADHD. Individual nutritional recommendations on the basis of the elimination of certain food items may become an additional therapeutic option for individuals with ADHD.
